# GLO GERM AND COVID-19: ILLUMINATING HYGIENE AND PROMOTING TRANSMISSION AWARENESS

**DOI:** 10.21010/Ajidv18i2S.1

**Published:** 2024-07-04

**Authors:** ZATLA Ilyes, BOUBLENZA Lamia, LEMERINI Wafaa, TRIQUI Chahinez

**Affiliations:** 1 Laboratory of Microbiology applied to the Food Industry, Biomedical and the Environment, Faculty of Natural and Life Sciences, Earth and Universe Sciences. Department of Biology. University of Tlemcen, Algeria; 2 Laboratory of Organic Chemistry, Natural Substances analysis, Faculty of Natural and Life Sciences, Earth and Universe Sciences. Department of Biology. University of Tlemcen, Algeria; 3 Laboratory of Physiology, Physiopathology and Biochemistry of Nutrition, Department of Biology, Faculty of Natural and Life Sciences, Earth and Universe, University of Tlemcen, Algeria

**Keywords:** Surface cleaning, detergents, hygiene, awareness, Glo Germ, COVID-19

## Abstract

**Background:**

Maintaining effective surface hygiene and preventing contamination is of paramount importance. Our study introduces Glo Germ, a versatile product available in various forms, which possesses the unique ability to reveal hidden truths under ultraviolet light, enhance understanding of hygiene, and spread awareness of COVID-19 transmission and preventive measures.

**Materials and Methods:**

A comprehensive study was conducted to assess different surface cleaning techniques’ effectiveness. Glo Germ, containing a fluorescent dye activated by ultraviolet light, was used to visualize germ spread and compare disinfectant cleaners’ efficacy. The study encompassed diverse surfaces and materials, aiming to identify optimal cleaning techniques for each context. Furthermore, a small illustrative study was conducted during a COVID-19 awareness presentation involving students. Glo Germ was applied to hands, revealing its subsequent spread to faces and surfaces. This visual experiment effectively emphasized hand hygiene and mask-wearing importance.

**Results:**

Results indicated that while water alone achieved satisfactory cleaning results, using detergent and the appropriate cleaning tools further improved efficacy. Notably, adhering to consistent patterns and applying pressure during cleaning proved essential. The student demonstration showed how contaminants spread quickly, highlighting hand hygiene’s significance and the potential extent of contamination through sneezing.

**Conclusion:**

Glo Germ inclusion in these experiments highlights its potential in educating about surface cleaning and microbial transmission, offering an interactive and engaging approach to promoting personal hygiene and fostering illness prevention awareness.

## Introduction

In the pursuit of public health and safety, maintaining impeccable surface hygiene and preventing contamination play pivotal roles. Establishing and adhering to appropriate cleaning and disinfection protocols are crucial steps in this endeavor. Regular cleaning with water or detergent, combined with the correct utilization of cleaning techniques and tools for diverse surfaces and areas, forms the foundation of effective surface hygiene practices (Pittet, 2001; Dancer, 2004).

Glo Germ is a commercial product available in liquid, gel, or powder form. What sets it apart is its remarkable ability to reveal hidden truths of contaminants under the magic of ultraviolet light. This powerful product contains a fluorescent dye that illuminates and brings to light the effectiveness of hand washing and surface cleaning practices (Glo Germ Company).

Our study aimed to compare the efficacy of different disinfectant cleaners and techniques applied in surface cleaning to combat germs, and while it is not a disinfectant itself, it serves as an invaluable educational tool. By analyzing the results, the study sought to identify the most effective disinfectant cleaners and cleaning techniques for different surfaces and materials. We also utilized this unique product to enhance understanding and raise awareness about the transmission of COVID-19, as well as the importance of preventive measures**.**

## Materials and Methods

**Figure 1:**
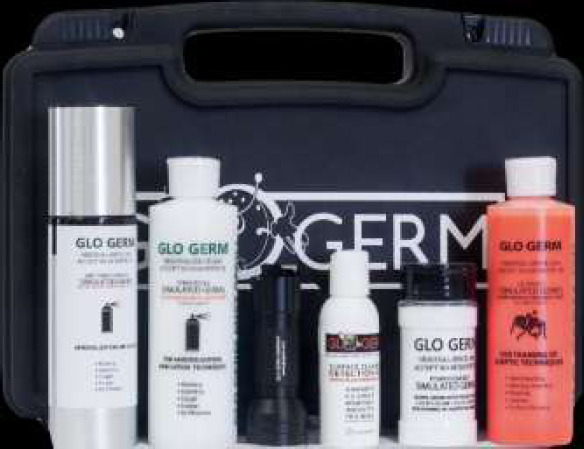
The Glo Germ Products full Kit.

A comprehensive visual study was conducted to evaluate the effectiveness of various surface cleaning techniques with cleaning tools in preventing the spread of germs using Glo Germ Kit (Figure 1). This last is a commercial product, containing a fluorescent dye activated by ultraviolet light. Two distinct types of surfaces, refined and coarse were employed. Also, both water and commercial detergent, as well as a smooth and rough cleaning cloth were applied to visualize and measure the efficacy of our cleaning habits. Furthermore, an illustrative study was undertaken during a COVID-19 awareness presentation, involving a random selection of students who were provided with Glo Germ cream and powder before the conference. Glo Germ was applied on their hands to follow its spread to surrounding objects, also the powder form of the product was scattered on one surface to observe the contamination of other areas. We also tried to reproduce an infected person’s sneeze pattern, to observe the aerosol dispersal, and to demonstrate the importance of wearing a face mask with the mist formula of our product.

### Ethical Considerations

This study adhered to ethical principles and guidelines to ensure the well-being, dignity, and privacy of the participants. Informed consent was obtained from all individuals involved in the visual experiment conducted during the COVID-19 Awareness Day: “Prevention & Vaccination”.

## Results and Discussion

In the study evaluating surface cleaning techniques, the results revealed that while using detergent helped remove the liquid product from both types of surfaces, water alone proved to be efficient enough in achieving satisfactory cleaning results. However, it should be noted that using the rough sponge was more effective in removing the product compared to the smooth one. Moreover, certain cleaning practices played a significant role in the effectiveness of the cleaning process. Following a consistent pattern while cleansing, avoiding back-and-forth movements, and applying pressure and friction were found to be essential in completely scraping off the product from the applied surfaces. The use of water alone, although effective to some extent, was unable to completely remove the fluorescent gel from the surfaces. These results, like those of other studies, highlight the importance of considering the cleaning products and tools used, the specific type of surface being cleaned, and the movement and pressure applied during the cleaning process (Lücke and Skowyrska, 2015).

**Figure 2:**
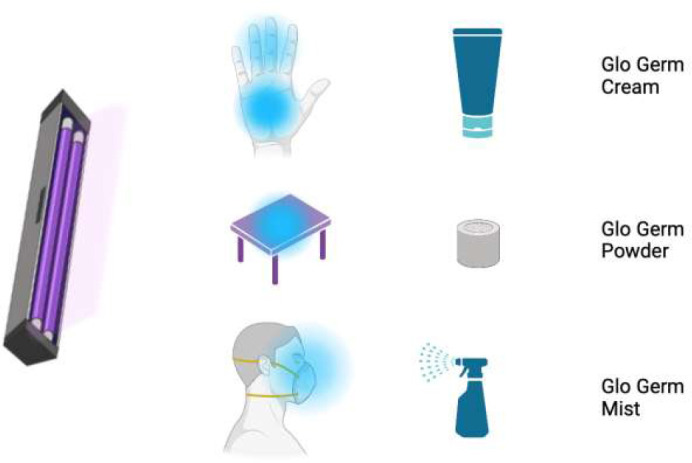
Illustration of the visual experiment products at the COVID-19 Awareness Day.

In the demonstration involving the students (Figure 2), the application of Glo Germ gel on their hands resulted in its widespread presence on their faces, phones, and clothes, even though it was initially applied only to their hands. This highlighted the ease with which contaminants can spread from one object to another, emphasizing the importance of hand hygiene in preventing the transmission of pathogens (Al Kadi and Salati, 2012 ; Zatla *et al.*, 2023a).

Additionally, the visualization of the sneeze pattern on the face, mouth, and clothes of the students illustrated the potential extent of contamination that can occur through sneezing and showed the importance of self-protect using a face mask specifically for the pandemic of COVID-19 (Zatla and Boublenza, 2023 ; Zatla *et al.*, 2023b). Furthermore, the scattering of powdered Glo Germ on surfaces resulted in the staining of other clean surfaces upon contact, demonstrating the ease with which germs and contaminants can transfer and adhere to different areas. This visual experiment effectively showcased the importance of hand hygiene and the significance of wearing masks, the students gained valuable insights into the potential consequences of an infected person sneezing and the importance of preventive measures.

## Conclusion

The inclusion of Glo Germ in these experiments has proven to be immensely valuable in the contribution to enhancing surface hygiene practices, strengthening infection control measures, and effectively curbing the spread of illnesses. The insights gained from these studies provide individuals with a better understanding of the efficacy of different cleaning techniques, the impact of cleaning products and tools, and the importance of proper cleaning protocols. It has also demonstrated its effectiveness as a captivating and engaging tool for educating individuals about the routes of microbial transmission, with a specific focus on respiratory viruses such as SARS-CoV-2. The interactive nature of the Glo Germ experience allows participants to witness firsthand, how easily germs can spread and how individuals can become infected. This process serves as a powerful reminder of the crucial role hygiene practices play in preventing the transmission of illnesses. By incorporating Glo Germ into future research endeavors, public health personnel can continue to refine and improve strategies aimed at promoting a cleaner and healthier environment, as it offers a unique and memorable approach to promoting personal hygiene and fostering a culture of illness prevention.
